# An Integrative Architecture for a Sensor-Supported Trust Management System

**DOI:** 10.3390/s120810774

**Published:** 2012-08-06

**Authors:** Denis Trček

**Affiliations:** Faculty of Computer and Information Science, University of Ljubljana, Tržaška c. 25, 1000 Ljubljana, Slovenia; E-Mail: denis.trcek@fri.uni-lj.si; Tel.: +386-1-4768-918; Fax: +386-1-4264-647

**Keywords:** sensors, trust management, human agents, modeling and simulation, multidisciplinary research

## Abstract

Trust plays a key role not only in e-worlds and emerging pervasive computing environments, but also already for millennia in human societies. Trust management solutions that have being around now for some fifteen years were primarily developed for the above mentioned cyber environments and they are typically focused on artificial agents, sensors, *etc*. However, this paper presents extensions of a new methodology together with architecture for trust management support that is focused on humans and human-like agents. With this methodology and architecture sensors play a crucial role. The architecture presents an already deployable tool for multi and interdisciplinary research in various areas where humans are involved. It provides new ways to obtain an insight into dynamics and evolution of such structures, not only in pervasive computing environments, but also in other important areas like management and decision making support.

## 1. Introduction

We are facing the era of ubiquitous computing, where various computing devices will be performing a plethora of advanced services. These devices will range from basic ones like passive sensors and radio frequency identification tags to advanced autonomous and powerful computing systems. In such environments trust is an essential ingredient if services, provided by the aforementioned structures, are supposed to be used by humans, especially those that influence security and safety. On the other hand, trust is an essential ingredient that has influenced humans' interactions and relationships at their core already for millennia. Moreover, it is crucial for the economical prosperity of organizations and even states (one often cited example about the need for more trust in e-environments is given in [1]).

Trust management solutions in both these cases are emerging as an important tool to support humans in their decision making. In the first case, trust management methodologies are about supporting users in cyber environments, while in the second case trust management methodologies are about supporting humans when making decisions in their domestic, natural domain–human structures that span from personal relationships to various kinds of organizations. There exist now numerous trust management solutions that have been developed during the last decade. These solutions are mainly using principles that are typically of artificial intelligence (AI) origin and are focused on cyber domain. Therefore they do not mimic the way that humans reason about trust. This is not to say that they are useless–on the contrary. But it is the fact that they are tied to their own domain, and a complement is needed with focus on humans. So when it comes to trust and when linking the above mentioned domains, the following key issues arise and they have to be addressed:

- Trust management methodology is needed that would realistically model specifics of humans when it comes to trust and would be understandable to a wide number of users.
- An interfacing metric is needed for translations between AI domain and humans' reasoning domain.
- A framework is needed that would link sensors with trust management domain to further improve (typically orally) reported trust assessments.

The reasoning behind these statements is as follows. As to the first and the second statement, the AI based trust management methodologies (which will be referred to as traditional methodologies in the rest of the paper) depend typically on quantitative approaches. However, research done so far in many areas suggests that humans, when it comes to trust, reason in qualitative terms. But even if the cognitive process itself is not qualitative (and this is related to the second bullet above), the way that humans describe and exchange trust assessments is still typically qualitative with estimates being taken from an ordinal scale. Addressing now the third statement, it may be noted that trust has numerous manifestations, which may be language expressions, deeds (which most effectively reflect the trust reasoning behind one's decisions), and physiological manifestations of trust processes. As to physiological manifestations—even when someone is not revealing a word about her trust processes, or does not take any action, the related cognitive processes result in physiological responses that can be captured by elementary sensors (like those for measuring the skin conductance changes and temperature changes), or more advanced sensors structures (like those that capture brain activities).

This is where the main contribution of this paper comes in. It presents extensions to an anthropocentric trust management methodology called Qualitative Assessment Dynamics (QAD). This methodology uses operands and operators, which have a linguistic background and are therefore understandable to the majority of users. By being of such nature, QAD can be also supported by sensors that could provide additional inputs for evaluation of trust assessments by measuring psychological and physiological responses of human agents (this is not the case with other trust management methodologies). Therefore the paper starts with a high-level scenario of such deployment in Section 2. In the same section, a holistic model for trust management is presented as well, which identifies areas that can be supported by sensors. With this background, Section 3 of this paper gives a brief analysis of the main trust management methodologies and identifies those areas that require further addressing—it turns out that the main issue is lack of human-centric methodologies. QAD fills this gap, and it is currently the only candidate methodology to deploy sensors for additional evaluations of trust assessments. On this basis, the fourth Section starts with an analysis of applicability of the main sensor based approaches that are used for dealing with cognitive processes (where trust belongs to). Afterward a concrete application with video sensors and QAD focused on facial expressions evaluations with all technical details for implementation is given; to the best of our knowledge, this is the first such approach presented in the literature. At the end, there is a conclusion that is followed by acknowledgements and references.

## 2. Trust, Sensors and Contexts

This section gives a refined model, which is focused on trust management and will serve to pinpoint those areas where sensors are to be deployed. It starts by giving brief technological basics of sensors to support and better present the logic and the main ideas behind the model that follows afterward.

Getting to sensors, the lowest level is mostly determined by pure sensors, which, on the basis of various phenomena tied to the behavior of semi-conductors, capture data about observed physical and physiological phenomena. After the sensing is done, the captured data has to be transferred for further processing. It may be transferred in a raw, analog form (which is rarely the case), or in a digital form. Once in a digital form, computer communication protocols start taking care of them. At the lowest end of the spectrum these are air-interfaces like those for near-field communication, NFC [2], continuing with bus protocols, where the most notable representatives are I^2^C that has been developed by Philips [3], and SPI that has been developed by Motorola [4].

**Figure 1.**
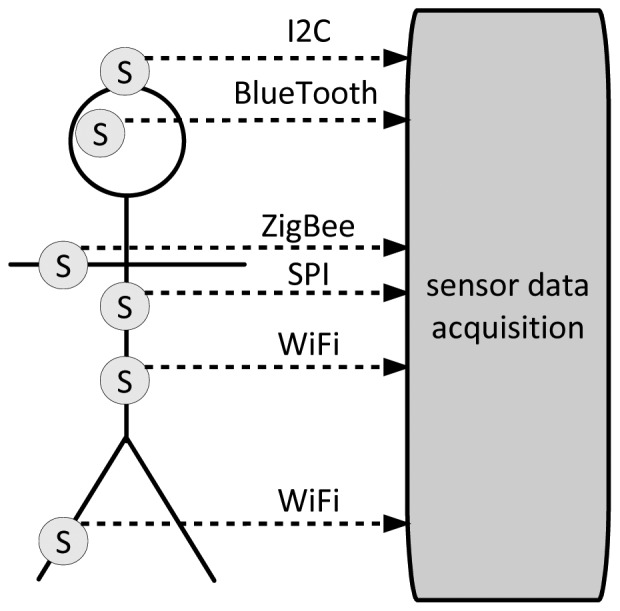
Sensors and trust related data capture.

Moving on to full blown communication protocols, which can be deployed in environments where sensors are backed up by microprocessors or microcontrollers, standards have already emerged for point-to-point and also full IP connectivity. A notable example for the first kind of connectivity is physical and link layers focused standard IEEE 802.15.4 [5], while the most notable example for sensor-adjusted IP networking is 6LoWPAN [6]. Consequently, as sensors are made accessible through wireless networks, security issues have become crucial, and these are already being a matter of research, which is very important for trust management purposes (see examples in [7,8]). Summing up, the technological reality has already made such advancements that we can focus on trust management support model and methodology, because technological problems about acquiring and transferring sensor data have already been solved to a large extent (see Figure 1).

Getting now to trust-for the time being, let us treat it informally as a kind of an assessment that may exist implicitly or may be explicitly expressed, and let it be denoted as α. Let us also assume that when referring to an agent, human-like agents or humans themselves will be meant. Now based on research described in [9], and as consequently addressed in [10,11], trust is bound to the following important factors:

- Rationality and irrationality. An agent's trust is driven by rational and irrational factors.
- Action binding. Trust presents a potential for an agent's deeds, *i.e.*, ways of interaction with the environment.
- Feed-back dependence. Trust is not a result of an independent mind, but it is influenced by the agent's environment. And when an agent is, e.g., forced to behave in a certain way, this may change her opinion about this behavior. Therefore trust may trigger a certain action, but it is also adjusted based on the results of the action.
- Trust differentiation. Trust evolves into various forms due to linguistic ability of an entity to express trust, and perception capabilities of a targeting entity (of course, trust can be mediated in an intentionally communicated form). Therefore it exists in two forms: minor trust that is expressed and communicated, and major trust, which is personal, intimate trust.
- Time dynamics. An agent's relation towards the object/subject being trusted is a dynamic, time dependent relation, where time should be treated as an intrinsic variable.

**Figure 2.**
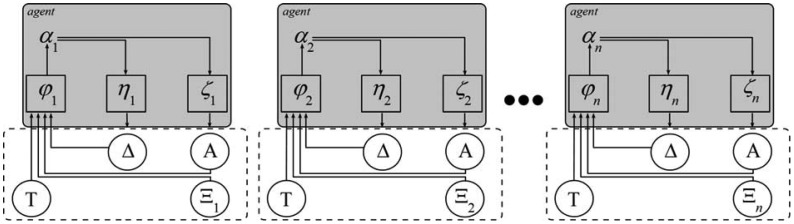
Sensor-focused trust management model.

Taking into account the above factors, a corresponding model is given in Figure 2. In this model T denotes the set of time increments *t*, Δ denotes the set of observed facts based assessments *δ* (*i.e.*, agent's physiological responses and deeds), and denotes the set of other agents' opinions. **A** denotes the set of assessments reported by agents. Thus *α_i_* (*t* + 1) = *φ_i_* (**A**(*t*), **Δ**(*t*), **Ξ***_i_*(*t*)) denotes the *i*-th agent major trust in step *t* + 1 that is calculated on the basis of **A**(*t*), **Δ**(*t*), **Ξ**(*t*), Further, the mapping from the set of major assessments to the set of deeds based assessments is defined by function *η: δ_i_* = *η_i_*(*α*). And finally, the expressed opinion *α* is modeled by function *ζ*, *i.e.*, *α_i_* = *ζ_i_* (*α*). It can be noted that trust forming factors are modeled by functions *φ*, *η* and *ζ*, as well as feed-back dependencies.

Comparing now Figures 1 and 2 it becomes evident where sensors come into play and what their role in supporting trust management is going to be. They will be indispensible to define or define or refine functions *ζ*, *η*, and *φ* and their outputs. All this is at the core of trust management.

## 3. From Traditional Trust Management Methodologies to QAD

There exist now various trust management methodologies that have been developed and proposed during the last ten to fifteen years. At the very beginning, these methodologies were not actually addressing trust, but security. However, it soon became clear that these two issues should not be traded one for another. When things became conceptually clear in the middle of the nineties, pure, trust focused methodologies emerged. The frequent basic area where they appeared was agents technologies. And within this area some typical trust management methodologies will be analyzed first, followed by an overview of trust management in sensors area.

1. In (intelligent mobile) agents area trust modeling and its management is mostly done by deploying artificial intelligence (AI) techniques:
  - One stream is based on Bayesian systems, where binary ratings are taken as inputs and where *a* denotes the number of positive ratings and *β* the number of negative ratings [12]. These ratings serve for computing reputation scores by updating beta probability density function $\text{BETA}(p|\alpha ,\beta )=\frac{\Gamma (\alpha ,\beta )}{\Gamma (\alpha ),\Gamma (\beta )}p^{a-1}{\left(1,p\right)}^{\beta -1},$ where 0 ≤ *p* ≤ 1, *α*, *β* > 0, and with the restriction *p* ≠ 0 if *α* < 1 and *p* ≠ 1 if *β* < 1. So the a posteriori reputation (*i.e.*, trust supporting) score is obtained on the basis of a priori reputation score that is updated with the new rating by taking into account the fact that *E*(*p*) = *^α^*/*_α_*_+_ *_β_* And this last result serves as a basis for probability expectation that the next interaction will be positive.
  - The second stream is based on advanced belief models, where the probability of an observed event being true and its complementary value (denoting the probability of the observed event being false) sum up to a value that is less than 1. In these models the remaining difference to 1 is interpreted as uncertainty. This stream builds on Dempster-Shafer Theory of Evidence, ToE. ToE starts with a set of possible states, called a frame of discernment Θ. Within Θ, exactly one state is assumed to be true at any time. Then a basic probability assignment, BPA, called also belief mass is defined. This is a function *m*: 2*^Θ^* → [0, 1], where with each sub-state *x* ∈ 2*^Θ^* a mapped value *m*(*x*) is associated, such that *m*(*x*) > 0, *m*(Ø) = 0, and $\sum_{x^{2\Theta }}m(x)=1$ (note that the belief mass expresses the belief assigned to the set *x* as a whole, and does not express any belief in subsets of *x*). On this basis, disbelief and uncertainty functions can be derived, so the whole triplet (*b,d,u*) is defined as follows:
(1) $$\begin{array}{rr} b(x)=\sum_{y\subseteq x}m(y), & d(x)\sum_{x\cap y=\varphi }m(y),u(x)=1-(b(x)+d(x)),\\ x,y\in 2^{\Theta } & \end{array}$$
    Having this triplet that represents trust of a certain agent it is now possible to introduce trust mimicking operators like consensus or recommendation, which is the case with Subjective logic [13].
  - The third stream is a bit different from the above ones and is based on game theory. One such proposed solution is personalized ranking systems, or PRS in short [14]. PRS system defines a relation *R* ⊆ *A* × *A* called ordering on set *A* if it is reflexive, transitive and complete. Further, a notation *L*(*A*) is introduced as the set of orderings on *A*, and it is denoted as ≤. Given these definitions, let $G_{V}^{S}$ be the set of all directed graphs *G* = (*V*, *E*) such that for every vertex *ν* ∈ *V*, there exists a (directed) path from *s* to *v*. Then a PRS is a functional where (for every finite vertex set *V* and every source *s* ∈ *V*) there exists mapping of every graph $G\in G_{V}^{S}$ to an ordering $\leq_{G,S}^{F}\in L(V)$.
2. Now as to sensors and trust management, it is often the case that sensors are treated as being analogous to agents. Therefore it should come as no surprise that the same arsenal of methodologies is also appearing in the sensors domain. One such example is given in [15], where ToE is deployed on top of behaviors of sensor nodes to obtain direct and indirect trust values. The above principles are refined in [16], where binary events-based trust supporting systems are extended, and Gaussian system is presented that serves for choosing appropriate peers for routing in wireless sensor networks. Trust supported routing is also in the focus of work presented in [17], where trust is based on measuring data and checking their spatial and temporal consistency. In [18] trust management solution is presented for fault-tolerant localization that uses reported (sampled) binary data to calculate trust index value for each sampling step. This index exponentially decreases with the step variable and each new value of this step variable depends on its previous value. In case of a faulty behavior, step variable results in increased exponent, which means that trust index is accordingly decreased. When a certain threshold is reached, the node is isolated.

The intention of the above overview is not to give an extensive coverage of all approaches that have appeared in trust management area recently. However, it presents some most important, specific or frequently cited approaches. Further, the above approaches certainly have many domains of application, but when it comes to human and human-like agents, certain issues still have to be addressed [14]: First, agents are not necessarily rational. Further, even if they are rational they (may) have problems probability. Further, even if they do not have problems with probability, many of them will not understand sophisticated mathematics like ToE. In addition, which is crucial for game-theoretic approaches, in case of trust they may have no preferences, and even if they have preferences, these may not be transitive.

QAD that has now been developed for almost ten years, was designed from scratch to address the above issues and to capture additional evidence data from the environment that have to be taken into account. And the latest technological advancements in sensors and communications areas enable such deployment, which will be elaborated in more detail in the text that follows.

Now the first thing that has to be resolved is a formal definition of trust. Trust clearly rests on cognitive processes inside our brains. What exactly these processes are at their core remains to be a matter of further research in the areas of psychology and neurosciences. But even not knowing the basic psychological or physiological drivers and trust forming factors trust can be abstracted at a level of its manifestation, regardless of the drivers behind this manifestation. And as to its manifestation, this may vary from simple, spoken expressions to complex signals observed through individual's behavior.

Thus, when talking about trust as an abstraction, which means actually treating trust at its face value, strong implications exist that it is sensible to treat trust as a relation. Therefore trust for our purposes is defined as follows: Trust is an assessment between entities *A* and *B* that is represented as a relation, which may be weighted quantitatively or qualitatively, and is denoted as *a_A,B_*, meaning agent *A*'s assessment of agent *B*. Having now a community with *n* agents, a corresponding assessment matrix **A** can be built. Rows in this matrix consist of certain agent's assessments about other agents in a community, while columns denote community's assessments towards observed agent. The assessments are taken from a five level (Likert like) ordinal qualitative scale that goes as follows: fully trusted (denoted as 2), partially trusted (denoted as 1), undecided (denoted as 0), partially distrusted (denoted as −1), and totally distrusted (−2). In addition, there exists a symbol “-”, which denotes values that are not expressed, be it intentionally or unintentionally.

It is often the case that agents take into account assessments of only certain members of the society when calculating new values, and ignore the rest of assessments (or ponder them). Therefore the dependency matrix **Ξ** is introduced with elements from the interval [0,1]. In its most basic mode of deployment, **Ξ** is used in a way where *ξ_i,j_* = 0 means that the corresponding element *a_i,j_* in **A** is excluded from trust assessment calculations, while *ξ_i,j_* = 1 means it is included. The first step in each calculation is application of **Ξ** to **A** to include only those operands that should be included, and only afterwards the remaining operands are subject to processing with the selected operator. Now as to operators, the following ones have been defined so far [14]:

-
$${⇑}_{i}:\text{max}(\alpha_{1,j}^{-},\alpha_{2,j}^{-},\alpha_{3,j}^{-},\ldots ,\alpha_{n1,j}^{-})\to \alpha_{i,j}^{+}\;i,j=1,2,\ldots ,n$$
-
$${⇓}_{i}:\text{min}(\alpha_{1,j}^{-},\alpha_{2,j}^{-},\alpha_{3,j}^{-},\ldots ,\alpha_{n1,j}^{-})\to \alpha_{i,j}^{+}\;i,j=1,2,\ldots ,n$$
-
$${↑}_{i}:\{\begin{array}{r} \alpha_{j,i}^{-}\to \alpha_{j,i}^{+}\\ \left\lfloor \alpha_{i,j}^{-}+1\right\rfloor \to \alpha_{i,j}^{+} \end{array}\begin{array}{c} \;if\frac{1}{n_{1}}\sum_{k=1}^{n_{1}}\alpha_{k,j}^{-}\leq \alpha_{i,j}^{-}\\ \;\text{otherwise} \end{array}$$
-
$${↓}_{i}:\{\begin{array}{r} \alpha_{i,j}^{-}\to \alpha_{i,j}^{+}\\ \left\lceil \alpha_{i,j}^{-}+1\right\rceil \to \alpha_{i,j}^{+} \end{array}\begin{array}{c} \;if\frac{1}{n_{1}}\sum_{k=1}^{n_{1}}\alpha_{k,j}^{-}\geq \alpha_{i,j}^{-}\\ \;\text{otherwise} \end{array}$$
-
$${↝}_{i}:\{\begin{array}{l} \left\lceil \frac{1}{n_{1}}\sum_{k=1}^{n_{1}}\alpha_{k,j}^{-}\right\rceil \to \alpha_{i,j}^{+}\\ \left\lfloor \frac{1}{n_{1}}\sum_{k=1}^{n_{1}}\alpha_{k,j}^{-}\right\rfloor \to \alpha_{i,j}^{+} \end{array}\begin{array}{c} \;if\frac{1}{n_{1}}\sum_{k=1}^{n_{1}}\alpha_{k,j}^{-}<0\\ \;\text{otherwise} \end{array}$$
-
$${↔}_{i}:\{\begin{array}{l} \left\lceil \frac{1}{n_{1}}\sum_{k=1}^{n_{1}}\alpha_{k,j}^{-}\right\rceil \to \alpha_{i,j}^{+}\\ \left\lfloor \frac{1}{n_{1}}\sum_{k=1}^{n_{1}}\alpha_{k,j}^{-}\right\rfloor \to \alpha_{i,j}^{+} \end{array}\begin{array}{c} \;if\frac{1}{n_{1}}\sum_{k=1}^{n_{1}}\alpha_{k,j}^{-}>0\\ \;\text{otherwise} \end{array}$$
-
$${↕}_{i}:\text{rand}(-2,-1,0,1,2)\to \alpha_{i,j}^{+}\begin{array}{c} \;i,j=1,2, \end{array}\ldots ,n$$
-
$${⊙}_{i}:\alpha_{i,j}^{-}\to \alpha_{i,j}^{+}\begin{array}{c} \;i,j=1,2, \end{array}\ldots ,n$$

A closer look at these operators reveals that they model some typical forms of human reasoning–their names go in turn as follows: extreme optimistic assessment, extreme pessimistic assessment, moderate optimistic assessment, moderate pessimistic assessment, centralistic consensus seeker assessment, non-centralistic consensus-seeker assessment, self-confident assessment and assessment-hoping. Finally, if an assessment is not disclosed (which means it is denoted by “−”) it keeps this value also after operation (pre-operation values are denoted by superscript “−”, and post-operation values by superscript “+”).

**Figure 3.**
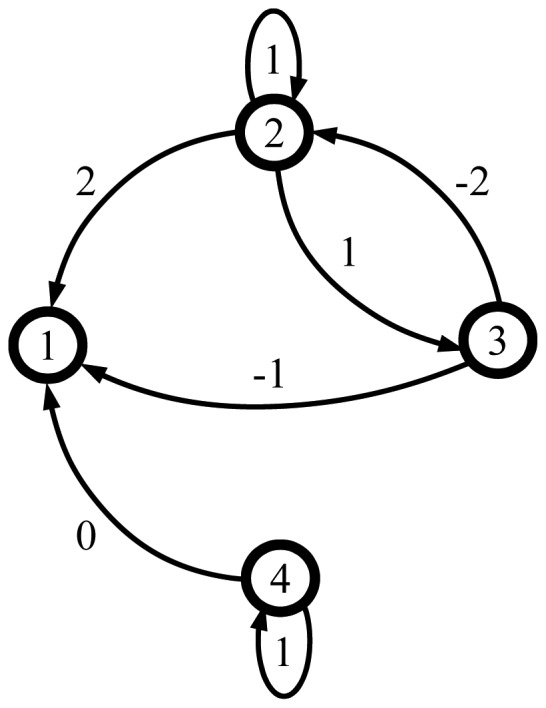
An example society graph with the corresponding assessment and dependency matrices.

An example of some society is given in Figure 3, where this society is presented with the graph and the corresponding assessment matrix **A** together with the dependency matrix Ξ:

Suppose now that agent 2 is governed by extreme optimistic assessment operator, agent 3 by centralistic consensus seeker and agent 4 by extreme pessimistic assessment operator. The society, as given in Figure 3, now makes a transition and its new state is given by the following matrix **A**:

$$\left[\begin{array}{cccc} - & - & - & -\\ 2 & 1 & 1 & -\\ 1 & 1 & - & -\\ -1 & -2 & - & 1 \end{array}\right]$$

To demonstrate QAD use, let us study a society with hundred agents. Initial assessments are such that all agents are undecided about other agents initially. Now 90% agents are initially governed by extreme optimistic operator, while 10% by extreme pessimistic operator. To obtain one possible dynamics of this society, let in each step 10% agents change their operator randomly; these agents are randomly chosen as well (newly assigned operators are equally likely).

Running now thirty simulation runs on this society, each of them taking forty-five steps, the histogram in Figure 5 is obtained (during this simulation, all assessments were taken into account by all agents in every step, *i.e.*, **Ξ** = [1]).

When elaborating the above results, one surprisingly finds out that an initially completely benevolent society (where agents did not care about one another) turned into a society where notable extremist assessments appeared–almost one third of all assessments became totally distrusted.

## 4. Integrating Sensors and QAD—A Concrete Technical Deployment

Trust is a result of cognitive processes and these processes may take part in (neo)cortical or sub-cortical parts of human brains [19]. The main approaches that enable study of these processes are strongly tied to sensors and they are the following ones:

- Electroencephalography, or EEG [20]—activities in neurons in brains are based on (ionic) currents and they result in varying voltage signals over the human scalp. Therefore sensors (electrodes) are placed on the scalp to capture these manifestations and analyze them (amplitudes are in the range of few micro volts, while the typical observed time resolution is in the range of milliseconds). EEG device costs are in the range of 10^5^ EUR, while there exist portable implementations in the price range of 10^4^ EUR. The latter kind of implementations could be used for trust management solutions, but there exist currently fundamentally unresolved issue and this is – how is trust reflected and recognized in EEG signals?
- Functional nuclear magnetic resonance or fNMR [21]—brain activities are (implicitly) acquired through changes in blood flow, detected by fNMR that captures blood-oxygen level dependent contrasts. Therefore neural activities are reflected in the change of blood flow by detecting oxygen-rich *vs*. oxygen-poor blood magnetization changes. These changes are observed in brain images with a delay of a few seconds. Typical NMR scanners costs, which are physically large devices, are in the range of 10^6^ EUR. This makes fNMR practically unsuitable for trust management support. In addition, the subject has to be completely still during investigation (typically 20 to 40 minutes). Not to mention that the basic unresolved issue is the same as stated above–how is trust reflected and recognized in fNMR images?
- Functional near-infrared spectroscopy or fNIRS [22]—NIRS as a spectroscopic method uses near-infrared band of electromagnetic spectrum (between 88 nano meters and 2500 nano meters). Using this band, NIRS devices measure molar absorptivity of emitted radiation by chemical species/human cells and on this basis monitor local tissue oxygenation. This oxygenation reveals the level of activity in a tissue. Typical NIRS devices costs are in the range of 10^4^ EUR and there exist portable implementations. This makes them, in principle (and to a limited extend) suitable for supporting trust management solutions. However, the main unresolved issue is the same as stated above for EEG anf NMR—how is trust reflected and recognized in fNIR images?
- Polygraphs [23]—these devices use elementary sensors to measure and record blood pressure, pulse, respiration, and skin conductivity. While the investigated subject is being questioned, the physiological responses are recorded that are used to differentiate between deceptive and non-deceptive answers. These devices cost in the range of 10^4^ EUR and are in principle portable.

Based on the above analysis, it follows that the first three approaches (EEG, fNMR, fNIR) are not suitable for wider integration with trust management solutions, so polygraphs, more precisely their core principles are chosen as appropriate, where (widely available) sensors are deployed for measuring blood pressure, skin conductivity, *etc*. In our case we will focus on facial expressions. For this purpose only one sensor is needed-a camera that is attached to a computer. The procedure goes as follows:

1. The subject that is providing assessments for matrix **A** is sitting in front of a computer with attached camera that captures her facial expressions.
2. The subject is given a question to get an oral assessment of her trust about another entity.
3. Afterward the examination procedure follows (an established procedure to evoke physiological responses is given in [24]), one question after another, which is administered over the network. With each question, subject's facial expressions are captured and stored in a database.
4. After the interrogation procedure is over, the obtained images are submitted to inference module that is trained to differentiate facial expressions. If the facial expressions obtained during the interrogation process do not match the provided assessment, the inference module adjusts the assessment value.

The technical details for the implementation of the above procedure are as follows (the below implementation provides core security services, *i.e.*, authentication, confidentiality, integrity, and non-repudiation [25]):

- The camera and the subject's PC are linked through IP (wired or wireless) network to the central premises of trust management system.
- The user and the camera are authenticated by deployment of public key algorithms through certificates and public key infrastructure. Further, camera is temper resistant, while the whole transfer of data to central premises is protected by IPSec [26].
- Once transferred to the central premises, pictures are stored in a database management system (e.g., MySQL), and a neural networks based inference system EMPATH [27] is used to analyze captured facial expressions [28,29] (neural networks as inference engines in this particular area are already well established method, and to date they have advanced to such extent that they are also entering the area of communications security [30]).
- Based on the results of EMPATH, directly obtained assessments in matrix **A** are modified accordingly (in the most extreme case, where facial expressions analysis leads to a dubious state, inference engine replaces the obtained assessment by “–”).

**Figure 4.**
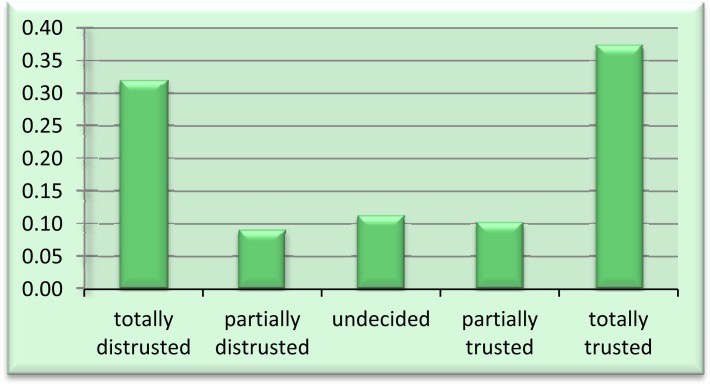
The complete sensor supported trust management architecture.

**Figure 5.**
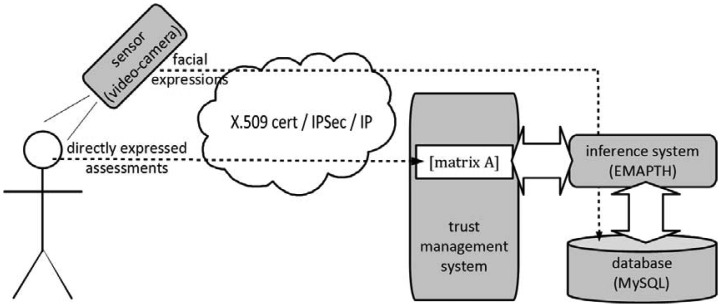
An example of application of QAD.

The whole architecture is presented in Figure 4. It is evident that for general applications wireless technology would be a preferred way for deployment, but this currently requires quite some issues to be resolved (one core problem is electromagnetic spectrum use, where an on-going research is focused on cognitive radio principles [31]).

One important issue has to be addressed before concluding this section. The core of the presented approach relies on deployment of polygraph principles. Actually, the main problem with polygraph- based methods is that they are not sufficiently (scientifically) sound enough to provide forensic evidence for legal investigation procedures [32], so it should be emphasized that this paper is not trying to convey the opposite message, but the reader should be also warned that this fact does not render the proposed procedure useless. On the contrary—when it comes to legal procedures and prosecution, evidence has to be “close to 100% certainty”. But with computationally supported trust management increasing accuracy of correctly obtained assessments for, e.g., 8% to 10% may present a significant improvement. Suppose one has to discern “the assessment noise” focused on only “trusted” and distrusted”, where 50% are reported “trusted” and 50% “distrusted”. If the investigator increases the accuracy, which shows that 60% assessments are actually trusted and 40% distrusted, this means an important step forward. However, more detailed elaboration of these issues, including details of investigation procedure, exceeds the scope of this paper and will be a matter of future research.

## 5. Conclusions

Trust in computing environments has now been on the research agenda for over a decade. It is interesting to note that the related solutions are intended for cyber environments and mostly based on established AI principles, which particularly holds true for agents' environments. This research paper, however, gives a complementary contribution based on methodology called Qualitative Assessment Dynamics, or QAD, and gives its extension. QAD is an anthropocentric methodology with operators and operands that have linguistic basis. Having such a background, it is not only understandable to a wide variety of users, but is also currently the only trust management methodology that enables integration with sensors when it comes to studying the core of trust phenomenon. Put another way, although there exist methodologies that address trust and sensors, these deal with trust management solutions adapted to sensors environments, and not with solutions that use sensors as a supportive element for evaluation of trust assessments, and trust management in general. No wonder, they are of different background, mostly deploying Bayesian probability based approaches—the approach in this paper, however, enables integration of sensors as mentioned above and this presents the first novelty of the paper.

Further, the paper gives a detailed way to implement the proposed architecture, which is its second novelty. It provides an analysis of the most important approaches to research of cognitive processes, where trust belongs to. These approaches are EEG, FNMR, fNIRT and polygraph. Based on this analysis it identifies concrete steps for the aforementioned deployment. Consequently, the presented anthropocentric methodology and architecture are integrated in a way that enables improved modeling and consequently understanding trust phenomenon dynamics in human structures. Together with other existing trust management methodologies the developed apparatus gives additional basis for further support of decision making processes that may span from simple tasks (e.g., buying low-cost goods or services in virtual environments) to complex and life threatening tasks (e.g., reporting patient's vital functions data through body area networks to central physician's location). And to the best of our knowledge this is the first paper of such kind of research in the literature.

## References

[1] Reding V. The Need for A New Impetus to the European ICT R & I Agenda.

[2] NFC Forum (2010). NFC Activity Specification, NFCForum-TS-Activity-1.0.

[3] Philips (2000). THE I2C-BUS SPECIFICATION.

[4] Motorola (2003). http://www.ee.nmt.edu/~teare/ee308l/datasheets/S12SPIV3.pdf.

[5] (2006). IEEE Wireless Medium Access Control (MAC) and Physical Layer (PHY) Specifications for Low Data Rate Wireless Personal Area Networks (WPAN), IEEE Standard 802.15.4.

[6] Rodrigues J.J.P.C., Neves P.A.C.S. (2010). A survey on IP-based wireless sensor network solutions. Int. J. Commun. Syst..

[7] Saleem S., Ullah S., Kyung S.K. (2011). A study of IEEE 802.15.4 security framework for wireless body area networks. Sensors.

[8] Kumar P., Lee K.J. (2012). Security issues in healthcare applications using wireless medical sensor networks: A survey. Sensors.

[9] Piaget J. (1999). Judgment and Reasoning in the Child.

[10] Trček D. (2004). Towards trust management standardization. Comput. Stand. Interfaces.

[11] Trček D. (2011). Trust Management Methodologies for the Web.

[12] Jøsang A., Ismail R., Boyd C. (2007). A survey of trust and reputation systems for online service provision. Decis. Support. Syst..

[13] Jøsang A. (2001). A logic for uncertain probabilities. IJUFKS.

[14] Tennenholtz M. Game-Theoretic Recommendations: Some Progress in an Uphill Battle.

[15] Feng R., Xu X., Zhou X., Wan J. (2011). A trust evaluation algorithm for wireless sensor networks based on node behaviors and d-s evidence theory. Sensors.

[16] Momani M., Chala S. Trust Management in Wireless Sensor Networks.

[17] Moya J.M., Vallejo J.C., Fraga D., Araujo A., Villanueva D., de Goyeneche J-M. (2009). Using reputation systems and non-deterministic routing to secure wireless sensor networks. Sensors.

[18] Xu X., Gao X., Xiong N. (2011). Trust index based fault tolerant multiple event localization algorithm for WSNs. Sensors.

[19] Pessoa L. (2008). On the relationship between emotion and cognition. Nat. Rev. Neurosci..

[20] Nunez P.L., Srinivasan R. (1981). Electric Fields of the Brain: The Neurophysics of EEG.

[21] Buxton R.B. (2002). Introduction to Functional Magnetic Resonance Imaging: Principles and Techniques.

[22] Madsen P.L., Secher N.H. (1999). Near infrared oximetry of the brain. Progr. Neurobiol..

[23] Abrams S. (1989). The Complete Polygraph Handbook.

[24] (2006). Federal Psycho-physiological Detection of Deception Examiner Handbook-Counterintelligence Field Activity Technical Manual.

[25] International Standards Organization (1989). Information Processing Systems-Open Systems Interconnection-Basic Reference Model, Security Architecture, Part 2.

[26] Kent S. (1998). Security Architecture for the Internet Protocol. IETF RFC 1825.

[27] Dailey M.N., Cottrell G.W., Padgett C., Adolphs R. (2002). EMPATH: A neural network that categorizes facial expressions. J. Cogn. Neurosci..

[28] Hess E.H. (1975). The role of pupil size in communication. Sci. Am..

[29] Hess E.H., How Your, Eyes Tell (1975). Hidden Thoughts. The Tell-Tale Eye.

[30] Koch R., Dreo G. (2009). Fast learning neural network intrusion detection system. Lect. Notes Comput. Sci..

[31] Marinho J., Monteiro E. (2012). Cognitive radio: Survey on communication protocols, spectrum decision issues, and future research directions. Wirel. Netw..

[32] Iacono W.G. (2001). Forensic ‘Lie Detection’: Procedures without scientific basis. J. Forensic Psychol. Pract..

